# Transcriptome and Metabolome Reveal Salt-Stress Responses of Leaf Tissues from *Dendrobium officinale*

**DOI:** 10.3390/biom11050736

**Published:** 2021-05-15

**Authors:** Mingze Zhang, Zhenming Yu, Danqi Zeng, Can Si, Conghui Zhao, Haobin Wang, Chuanmao Li, Chunmei He, Jun Duan

**Affiliations:** 1 Key Laboratory of South China Agricultural Plant Molecular Analysis and Gene Improvement, South China Botanical Garden, Chinese Academy of Sciences, Guangzhou 510650, China; zhangmingze@scbg.ac.cn (M.Z.); zhenming311@scbg.ac.cn (Z.Y.); zengdanqi20@scbg.ac.cn (D.Z.); cans2013@163.com (C.S.); zhaoconghui@scbg.ac.cn (C.Z.); wanghaobin17@scbg.ac.cn (H.W.); 2 College of Biological Science and Agriculture, Qiannan Normal University for Nationalities, Duyun 558000, China; 3 College of Life Sciences, University of Chinese Academy of Sciences, Beijing 100049, China; 4 Guangzhou Keneng Cosmetic Scientific Research Co., Ltd., Guangzhou 510800, China; lichm@danzi.cn

**Keywords:** *Dendrobium officinale*, salt stress, flavonoids, jasmonic acid, transcriptome, metabolome

## Abstract

*Dendrobium officinale* Kimura et Migo is a precious traditional Chinese medicine. Despite *D*. *officinale* displaying a good salt-tolerance level, the yield and growth of *D*. *officinale* were impaired drastically by the increasing soil secondary salinization. The molecular mechanisms of *D. officinale* plants’ adaptation to salt stress are not well documented. Therefore, in the present study, *D. officinale* plants were treated with 250 mM NaCl. Transcriptome analysis showed that salt stress significantly altered various metabolic pathways, including phenylalanine metabolism, flavonoid biosynthesis, and α-linolenic acid metabolism, and significantly upregulated the mRNA expression levels of *DoAOC*, *DoAOS*, *DoLOX2S*, *DoMFP*, and *DoOPR* involved in the jasmonic acid (JA) biosynthesis pathway, as well as rutin synthesis genes involved in the flavonoid synthesis pathway. In addition, metabolomics analysis showed that salt stress induced the accumulation of some compounds in *D. officinale* leaves, especially flavonoids, sugars, and alkaloids, which may play an important role in salt-stress responses of leaf tissues from *D. officinale*. Moreover, salt stress could trigger JA biosynthesis, and JA may act as a signal molecule that promotes flavonoid biosynthesis in *D. officinale* leaves. To sum up, *D. officinale* plants adapted to salt stress by enhancing the biosynthesis of secondary metabolites.

## 1. Introduction

*Dendrobium* is the second largest genus in the Orchidaceae family, which is the largest and most highly evolved family group among the angiosperms [1]. Among the *Dendrobium* species, *D. officinale* Kimura et Migo, commonly known as “Tiepi shihu”, is considered to have the best medicinal properties in traditional Chinese medicine (TCM) [2]. At least 190 compounds have been isolated from *D. officinale*, including polysaccharides, phenanthrenes, phenols, bibenzyls, essential oils, alkaloids, flavonoids, and several trace mineral elements [3,4]. Modern pharmacological studies have shown that *D. officinale* can enhance immunity, lower blood glucose levels, protect the liver and stomach, and has anti-tumor, anti-oxidation, anti-bacteria, and anti-inflammation functions [3,5].

Traditionally, stems of *D. officinale* are the main medicinal parts used in TCM, as they contain high amounts of polysaccharides [6,7]. However, during the harvesting period, a large number of leaves grow on the stems and contribute about 50% of the total biomass [8]. *D. officinale* leaves also contain many medicinal compounds. For example, they contain high contents of flavonoids, mainly rutin [8]. Rutin, known as vitamin P, can strengthen blood vessels, assist with the usage of vitamin C, assist with the production of collagen, and lower cholesterol levels, the occurrence of blood clots, and high blood pressure [9]. Therefore, comprehensive utilization of *D. officinale* leaves is among the great issues for related industrial requirements.

Salinity is a common abiotic stress and its effects are increasing rapidly on a global scale, severely limiting plant growth, productivity, and geographical distribution [10]. Generally, salt-sensitive plants are difficult to survive at 100 mM sodium chloride (NaCl) or higher. Conversely, salt-tolerant plants can grow at a concentration of NaCl greater than 250 mM [11]. Plants adapt to salt stress by transcriptional regulation and gene expression, facilitating the biosynthesis of metabolites, such as osmoprotectants and antioxidant compounds [12]. The biosynthesis of plant secondary metabolites can be induced under moderate and short-term environmental stress [13]. Researches showed that *D.* orchid responds to salinity by performing osmotic adjustment and sequestering Na^+^ or Cl^−^ in the roots, preventing their movement to the upper plant parts [14]. Nevertheless, the molecular mechanisms of *D. officinale* plant response to salt stress requires more investigation.

Transcriptome analysis is a powerful strategy for investigating the expression patterns of particular metabolite biosynthetic genes. The metabolites which directly reflect metabolic state of an organism or cell during a specific period of time can be detected by using metabolomics. Transcriptomics and metabolomics open a new avenue for the discovery of novel genes, the identification of gene function, and the detection of secondary metabolites which are responsible for plant adaptation to stress [15]. Therefore, in the present study, *D. officinale* plants were treated with 250 mM NaCl. Then, transcriptome and metabolome profiles of leaves were compared between the salt stress treatment and a control treatment to elucidate the molecular mechanisms mediating *D. officinale* plant salt-stress responses.

## 2. Materials and Methods

### 2.1. Plant Materials and Experimental Design

The *D*. *officinale* used in this study were provided by the Guangxi State-owned Huangmian Forest Farm (Liuzhou, Guangxi Zhuang Autonomous Region, China). In vitro-derived *D. officinale* plantlets, 8–9 cm in height, were subjected to half-strength (1/2) Murashige and Skoog (MS) [16] culture medium supplemented with 250 mM NaCl (Guangzhou Chemical Reagent Factory, Guangzhou, China), and then grown in an artificial climate chamber at a temperature of 25 °C, with a 16/8 (day/night) photoperiod for 0, 4, and 12 h. Leaves were sampled and quick-frozen with liquid nitrogen for RNA isolation. Isolated RNA was used for transcriptome detection.

Another group of plantlets under the same conditions as above were grown for 14 days, after which the leaves were used for nontargeted metabolomic analysis and to measure the contents of total flavonoids and anthocyanidin. Experimental control treatments consisted of *D. officinale* plantlets of similar size grown in 1/2 MS without 250 mM NaCl.

The third group of plantlets, which were about 10 cm in length, were grown in 1/2 MS supplemented with 250 mM NaCl for 0, 2, 6, 12, and 24 h, then leaf samples were collected and used to determine jasmonic acid (JA) content.

### 2.2. RNA Extraction and Sequencing

RNA was isolated from each sample with three independent biological replicates using a Biospin Plant Total RNA Extraction Kit (Hangzhou Bioer Technology Co. Ltd., Hangzhou, China) following the manufacturer’s instructions. The cDNA library preparations for next-generation sequencing were generated from 1 μg RNA of each *D. officinale* leaf sample using NEBNext^®^ Ultra^TM^ RNA Library Prep Kit for Illumina (NEB, Ipswich, MA, USA). Each cDNA library was purified with AMPure XP system (Beckman Coulter, Beverly, MA, USA) and its quality was assessed by using Agilent RNA 6000 Nano Kit on the Agilent Bioanalyzer 2100 system (Agilent Technologies, Santa Clara, CA, USA), then the cDNA libraries were sequenced and analyzed in Biomarker Technologies Co., Ltd. (Beijing, China). Based on sequencing-by-synthesis (SBS) technology, cDNA libraries were sequenced using the Illumina high-throughput platform. The cleaned reads generated by our cDNA libraries were mapped to the genome of *D. officinale* with HISAT2 software [17,18]. The transcripts with mapped reads were assembled using StringTie [19]. Fragments Per Kilobase of transcript sequence per Millions mapped reads (FPKM) was calculated to assess gene expression levels. The expression level of each gene was calculated with the DEseq2 package [20]. The genes with a fold change (FC) ≥ 2 and false discovery rate (FDR) < 0.01 by pairwise comparisons were defined as differentially expressed genes (DEGs). A Kyoto Encyclopedia of Genes and Genomes (KEGG) database (https://www.genome.jp/kegg/pathway.html, accessed on 1 March 2021) was used for KEGG pathway analyses. KEGG category enrichment with a *q* value (corrected *p* value) < 0.05 was considered significantly enriched. The protein–protein interaction (PPI) analysis was performed by STRING database (http://string-db.org/) and was visualized using Cytoscape [21]. The heat maps for DEGs were plotted by using TBtools [22]. Raw sequence reads can be downloaded from the website (https://www.ncbi.nlm.nih.gov/bioproject/?term=PRJNA715099, accessed on 1 March 2021).

### 2.3. Quantitative Reverse Transcription-PCR

The first-strand cDNA was performed with a GoScriptTM Reverse Transcription System (Promega, Madison, WI, USA) according to the manufacturers’ instructions. Quantitative reverse transcription-PCR (qRT-PCR) analysis was performed by Unique Aptamer^TM^ qPCR SYBR^®^ Green Master Mix (No Rox, Tianjin, China) with a LightCycler 480 system (Roche, Basel, Switzerland). The *D. officinale* actin (NCBI accession No. JX294908) served as an internal control. The 2^−ΔΔCt^ method was used to calculate the relative expression levels of genes [23]. The primers for qRT-PCR in this study were listed in Table S1.

### 2.4. Nontargeted Metabolic Profiling

To determine significantly changed metabolites (SCMs) between the control treatment and salt stress treatment, three biological replicates of leaves were analyzed. The leaf samples were delivered to Novogene Biotech Co., Ltd. (Beijing, China) to analyze the nontargeted metabolome. The *D. officinale* leaf samples (100 mg) were homogenized and were extracted with pre-chilled 80% methanol solution containing 0.1% (*v*/*v*) formic acid. After being incubated on ice for 5 min, the extracted samples were centrifuged at 15,000× *g*, 4 °C for 10 min. Then, the supernatant of each leaf sample was diluted to final concentration containing 53% methanol by liquid chromatography–mass spectrometry (LC–MS) grade water and was injected into the LC–MS/MS system analysis. LC–MS/MS analyses were performed using an ExionLC^TM^ AD system coupled with a QTRAP^®^ 6500+ mass spectrometer (both from SCIEX, Framingham, MA, USA). Equal volumes of each experimental sample were combined for quality control (QC) samples. Blank samples were 53% methanol aqueous solution containing 0.1% formic acid instead of experimental samples and were pretreated in the same way as the experimental samples. The high-performance liquid chromatography (HPLC) and MS conditions, as well as metabolite identification and quantification, were described as per by He et al. [24].

The identified metabolites were annotated using the KEGG database (http://www.genome.jp/kegg/, accessed on 1 March 2021), the HMDB (human metabolome database, http://www.hmdb.ca/, accessed on 1 March 2021), and the Lipidmaps database (http://www.lipidmaps.org/, accessed on 1 March 2021). The metabolites with the variable importance in the projection (VIP) > 1, *p* value < 0.05, and the absolute value of log_2_FC ≥ 1.3 were considered to be differential metabolites.

### 2.5. Measurement of Anthocyanidin, Total Flavonoid, and JA Content

Fresh leaf samples (0.2–0.5 g) were ground into powder in liquid nitrogen, then immersed in acidified methanol (0.3% HCl (*v*/*v*), 5 mL) at 4 °C for 24 h in the dark, and then centrifuged (5000× *g*) for 20 min at low temperature (4 °C). Supernatant was assayed for measurements of the total anthocyanidin by using an ultraviolet-visible spectrophotometer (UV-6000PC, Shanghai Metash, Shanghai, China) at 530 nm. A colorimetric method was used to analyze the total flavonoid content as described by Ren et al. [25]. Rutin (Chengdu Herbpurify Co., Ltd., Chengdu, China) solution was used as standard. The JA content in leaves was measured using a quantitative enzyme immunoassay kit (Meimian Biotech Co., Ltd., Yancheng, China). For measurement of anthocyanidin, total flavonoid, and JA content, each sample was replicated three times. ANOVA with a Student–Newman–Keuls test was performed in IBM SPSS Statistics (version 20.0 software for Windows; SPSS, Chicago, IL, USA) to analyze the data.

## 3. Results

### 3.1. Transcriptional Profiling

To study the system-wide changes in the *D. officinale* leaves under salt stress, three time points (0, 4 and 12 h after treatment) were selected for analysis. We obtained 67.78 Gb of clean data in total after mRNA sequencing of nine samples with at least 6.14 Gb of clean data for each sample (Table S2). In each sample, more than 93.27% of bases had a score of Q30 or above, indicating that the sequencing results could be used for subsequent analysis. The clean data were mapped to the reference genome [18], with the mapping ratio varying from 91.04% to 91.52% (Table S2).

**Figure 1:**
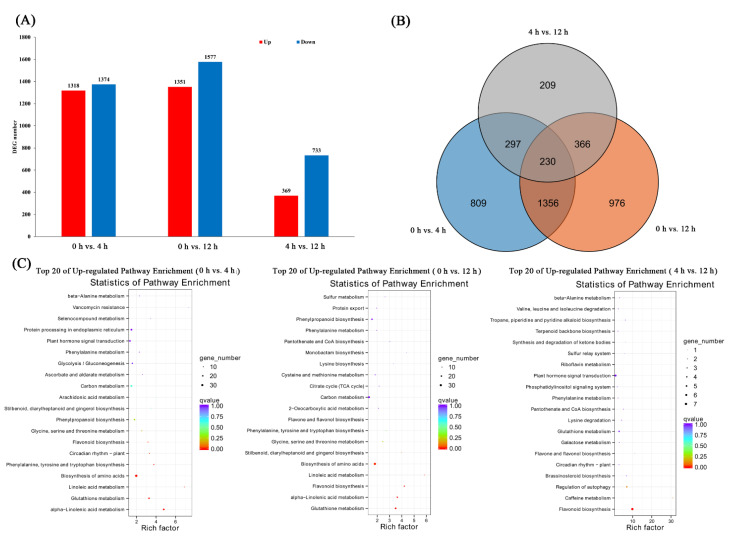
Differentially expressed genes (DEGs) at different time points under salt treatments. (**A**) Number of DEGs. (**B**) Venn diagram of DEGs. (**C**) Top 20 enriched pathways for overlapping salt responsive genes of 0 h vs. 4 h, 0 h vs. 12 h, and 4 h vs. 12 h.

For the pairwise comparisons of 0 h vs. 4 h, 0 h vs. 12 h, and 4 h vs. 12 h, 2692 (1318 upregulated; 1374 downregulated), 2928 (1351 upregulated; 1577 downregulated), and 1102 (369 upregulated; 733 downregulated) DEGs, respectively, were identified (Figure 1A). Of these DEGs, 1586 were the same in both 0 h vs. 4 h and 0 h vs. 12 h; 527 were the same in both 0 h vs. 4 h and 4 h vs. 12 h; and 596 were the same in both 0 h vs. 12 h and 4 h vs. 12 h. Additionally, 230 DEGs were the same in 0 h vs. 4 h, 0 h vs. 12 h, and 4 h vs. 12 h (Figure 1B). KEGG analysis showed that several metabolic processes, including plant hormone signal transduction, alpha-linolenic acid metabolism, phenylpropanoid biosynthesis, flavonoid biosynthesis, and flavone and flavanol biosynthesis were significantly enriched in DEGs under salt treatment (Figure 1C).

**Figure 2:**
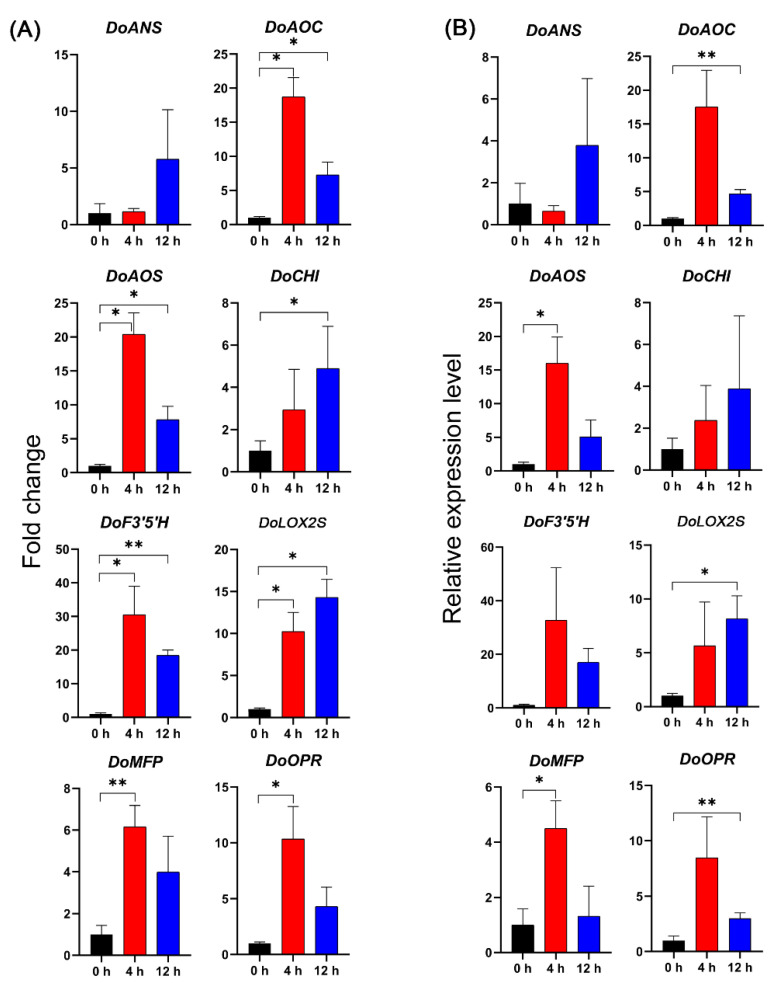
qRT-PCR confirmation of expression profiles obtained by RNA-Seq transcriptome analysis at different times after salt treatment. Fold-change in transcript abundance obtained by both qRT-PCR and RNA-Seq are presented on the same graph for eight representative encoding genes. Column (**A**) shows the fold-change in the Fragments Per Kilobase of transcript sequence per Millions mapped reads (FPKM) value obtained by RNA-Seq, and column (**B**) shows the fold-change in the expression determined by qRT-PCR. DoANS, DoAOC, DoAOS, DoCHI, DoF3′5′H, DoLOX2S, DoMFP, and DoOPR are anthocyanin synthase, allene oxide cyclase, allene oxide synthase, chalcone isomerase, flavonoid 3′,5′-hydroxylase, lipoxygenase, peroxisomal fatty acid beta-oxidation multifunctional protein, and 12-oxophytodienoate reductase, respectively, in *D. officinale*. Data represent means and SDs. Statistical significance was calculated using GraphPad Prism 8 (Version 8.0.2, GraphPad Software, La Jolla, CA, USA). * *p* < 0.05; ** *p* < 0.01.

Eight DEGs (three genes related to flavonoid synthesis and five genes related to JA synthesis) were selected for qRT-PCR analysis, and their expression patterns were consistent with the RNA-Seq results (Figure 2), indicating that the RNA-Seq data were reliable.

**Figure 3:**
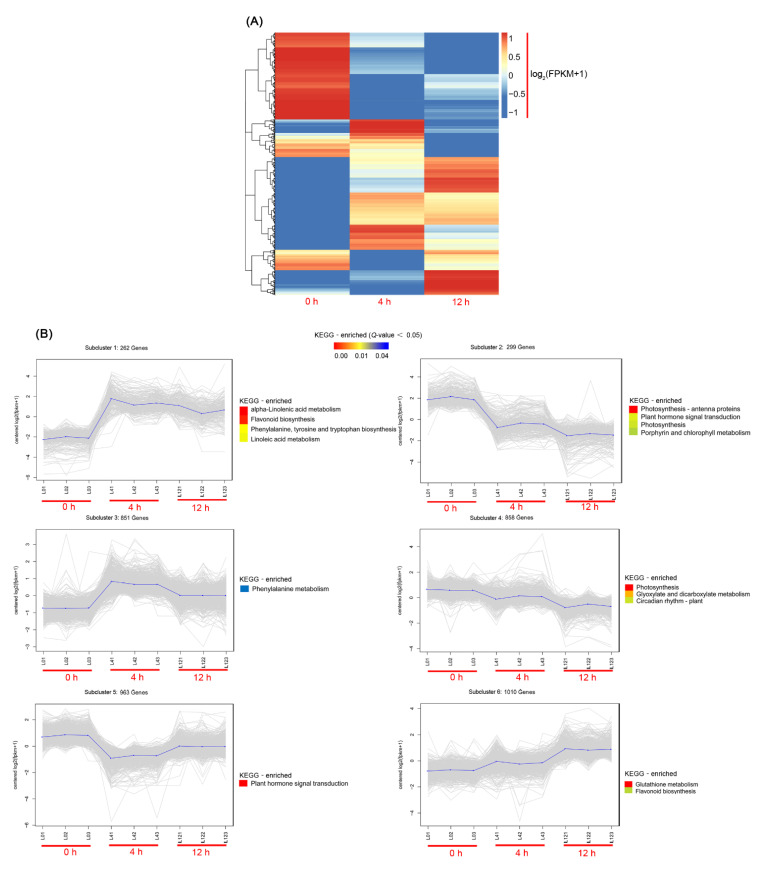
Heat map analysis of the overall gene expression profiles and Kyoto Encyclopedia of Genes and Genomes (KEGG) category distribution of the DEGs in the six expression subclusters composing the transcriptome of *D. officinale* leaves under salt stress. (**A**) The hierarchical analyses of co-expression transcripts at different time points of salt stress. Blue indicates the downregulated and red indicates the upregulated expression of genes. Expression values were Z-scaled (log_2_(FPKM+1)). (**B**) Six subclusters of expression patterns and KEGG enrichment categories. Expression values were Z-scaled. The blue line represents the average expression value of the members at each time point. The degree of enrichment of categories in each cluster is shown in different colored boxes. Red represents the over-represented categories and blue represents the under-represented categories. KEGG category enrichment was computed using the Cluster package and *q* value package (*q* value < 0.05) in R.

Significant changes in KEGG pathways were identified using DEG co-expression analysis. To decipher the general trend in gene expression profiles, we subjected the 6722 DEGs to *k*-means clustering analysis. The 6722 DEGs were hierarchically clustered (Figure 3A) into six subclusters (Figure 3B). KEGG analysis was performed for DEGs belonging to each cluster with a *q* value < 0.05. According to subcluster 1, 3 and 6, salt stress induced the synthesis of secondary metabolites in *D. officinale* leaves. The α-linoleic acid metabolic pathway is involved in the synthesis of JA. The expression levels of related genes in this pathway were significantly enriched after salt stress treatment, as shown in subcluster 1 (Figure 3B).

**Figure 4:**
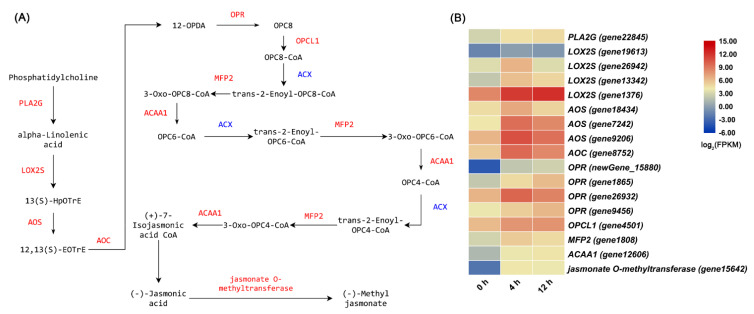
The jasmonic acid (JA) biosynthetic pathway and related upregulated genes under salt treatment. (**A**) The JA biosynthetic pathway and its associated enzymes. (**B**) Heat map analysis of upregulated gene expression in the JA biosynthetic pathway under salt treatment.

Many genes involved in JA biosynthesis were upregulated under salt stress (determined by checking the detailed FPKM values of these DEGs, Figure 4). Under salt stress, genes involved in photosynthesis were significantly downregulated in subcluster 2 and 4, but genes related to glutathione metabolism were significantly upregulated in subcluster 6 (Figure 3B).

Transcription factors (TFs) are considered to be the most important regulators controlling gene expressions. A total of 31 *bHLHs* (10 upregulated and 21 downregulated), 29 *MYBs* (8 upregulated and 21 downregulated), 17 *WRKYs* (7 upregulated and 10 downregulated), 110 *RLK-Pelles* (53 upregulated and 57 downregulated), 22 *NACs* (11 upregulated and 11 downregulated), and 49 *AP2/ERFs* (19 upregulated and 30 downregulated) were differentially expressed under the salt stress treatment compared with under the control treatment (Figure S1). These differently expressed TFs may be critical genes in responses to salt stress.

Transcriptome data analysis showed that the expression level of *DobHLH* (*gene9655*) was significantly downregulated under salt stress (Figure S2A). Putative protein DoTIFY10As (gene3248 and gene3249) and DoTIFY10Bs (gene20757, gene28238, and gene13335) that interact with protein DobHLH (gene9655) were identified by blasting in the STRING database (Figure S2B). The sequence of protein DobHLH (gene9655) was blasted in the Plant Transcription Factor Database (http://planttfdb.gao-lab.org/blast.php). It was found that DobHLH (gene9655) was highly homologous with *Arabidopsis thaliana* MYC4 (AtMYC4) (Table S3). In addition, AtMYC4 interacts with some proteins in the JA ZIM-domain protein (JAZ), F-box protein CORONATINE INSENSITIVE1 (COI1), and TIFY transcription factor families (Figure S2C). Transcriptional expression profiles showed that the expression levels of some *DoTIFY10As* and *DoTIFY10Bs* were upregulated under salt stress (Figure S3). Therefore, it is speculated that DobHLH (gene9655) may also interact with some proteins in the JAZ, COI1, and TIFY transcription factor families in *D. officinale*. Furthermore, the expression level of *DoMYB* (*gene6015*) was significantly upregulated under salt stress (Figure S4A). Thirteen proteins that interact with DoMYB (gene6015) were obtained in the STRING database (Figure S4B). It was found that DoMYB (gene6015) was most homologous with *A. thaliana* MYBTT2 (AtMYBTT2, Alias: AtMYB123) by blasting its protein sequence into the Plant Transcription Factor Database (Table S4 and Figure S5). In addition, AtMYBTT2 interacts with AtDFR and AtF3H proteins (Figure S4C). Therefore, it is speculated that DoMYB (gene6015) may also interact with DFR and F3H proteins in *D. officinale*.

### 3.2. Metabolomic Profiling and DEGs in Related Pathways

To explore the differences in metabolite compounds between the control treatment and salt-stress treatment, metabolomic profiling of tissue-cultured *D. officinale* plantlets after 14 days of treatment with or without 250 mM NaCl was performed using an untargeted metabolomics method. The results of principal component analysis (PCA) showed that PC1 (75.5%) and PC2 (8.17%) effectively separated the control samples from the saline stress samples (Figure S6A). Compared with the control group, the types of metabolites with increased content under salt stress were more than those of metabolites with decreased content (Figure S6B). A comparison between SCMs of the control and salt-stress treatments showed that the following pathways were enriched: phenylalanine metabolism, biosynthesis of flavone and flavanol, isoflavonoid biosynthesis, biosynthesis of amino acids including leucine and isoleucine, biosynthesis of secondary metabolites, and alpha-linolenic acid metabolism (Figure S6C).

**Figure 5:**
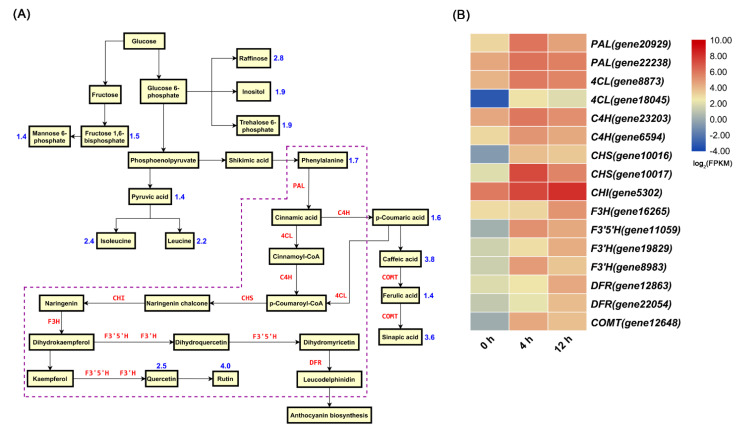
Sugar, amino acid, and flavonoid metabolic pathways and related upregulated genes under salt stress. (**A**) Sugar, amino acids, and flavonoid metabolic pathways. Blue text shows the value of log_2_fold change (FC) in metabolites under salt stress (see Table S5 for details). (**B**) Heat map analysis of upregulated gene expression under salt stress.

The contents of many compounds increased under salt stress, especially that of flavonoids (Figure 5A). This change trend is consistent with that of the upregulated expression of genes involved in flavonoid biosynthesis under salt stress (Figure 5B). The increasing content of rutin in leaves is likely attributed to the constant and high expression of these genes under salt stress.

**Figure 6:**
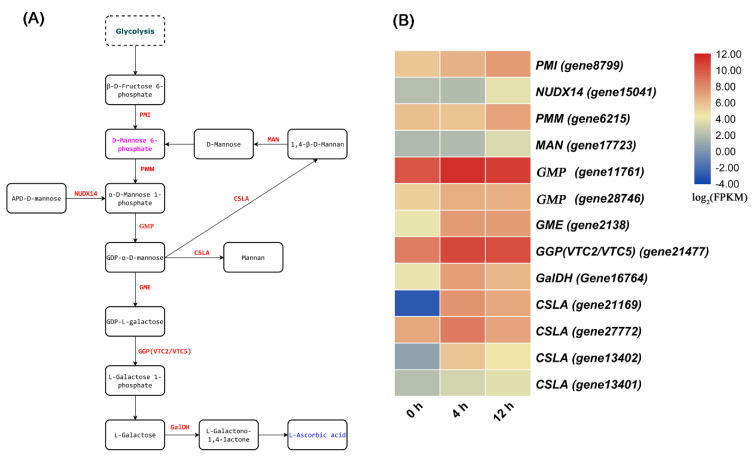
The ascorbic acid biosynthesis pathway and related upregulated genes under salt treatment. (**A**) The ascorbic acid biosynthesis pathway. (**B**) Heat map analysis of upregulated gene expression under salt treatment.

**Figure 7:**
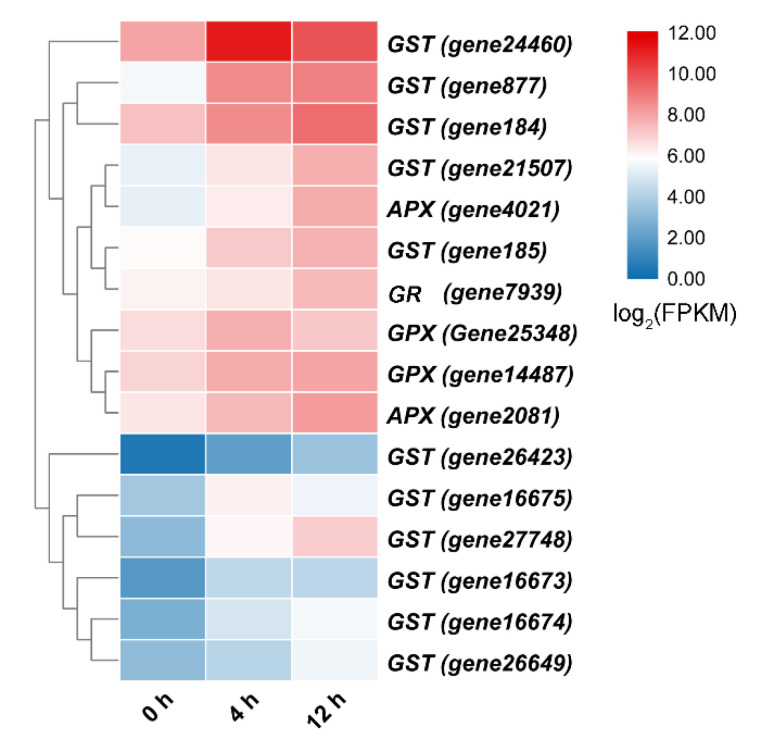
Heat map analysis of upregulated gene expression associated with the ascorbate-glutathione (AsA-GSH) cycle under salt treatment.

In addition, salt stress induces the accumulation of other secondary metabolites (Table S5). For example, some primary metabolites such as raffinose, mannose 6-phosphate, and trehalose 6-phosphate (T6P) showed accumulation under the salt treatment compared with under the control treatment (Figure 5A). Moreover, ascorbic acid (AsA) was detected under salt stress (Figure 6A), but its content did not differ significantly compared with that under the control treatment (Table S5). However, the expression levels of the genes encoding GDP-d-mannose pyrophosphorylase (GMP), GDP-d-mannose-3′,5′-epimerase (GME), and GDP-l-galactose phosphorylase (GGP) significantly increased under salt stress (Figure 6B). In addition, the expression level of gene encoding L-galactono-1,4-lactone dehydrogenase (GLDH) did differ significantly between the two treatments, which reflects the results observed for AsA. Furthermore, the expression levels of genes involved in ascorbate-glutathione (AsA-GSH) cycle were significantly upregulated under salt stress (Figure 7).

### 3.3. Salt Stress Enhanced the Contents of Total Flavonoids, Anthocyanidin, and JA

**Figure 8:**
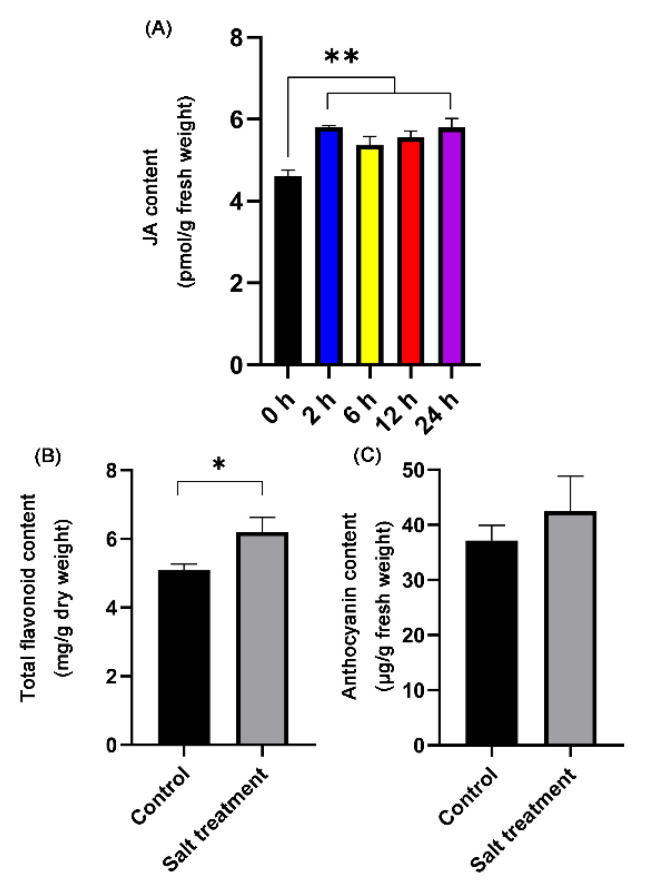
The contents of JA, total flavonoids, and anthocyanidin in leaves under salt stress. (**A**) JA content at different time points under salt stress. (**B**) The contents of total flavonoids in control and salt-stress treatments. (**C**) The contents of anthocyanidin in control and salt-stress treatments. Statistical significance was calculated using Student’s *t*-tests. * *p* < 0.05, ** *p* < 0.01.

The content of JA was significantly higher at 2 h compared with 0 h after salt stress, but then only differed slightly between 2 h and 24 h after salt stress treatment (Figure 8A). Compared with the control treatment, the total flavonoid content was significantly higher under the salt stress treatment (Figure 8B). However, the anthocyanidin content was only marginally different between the control treatment and the salt stress treatment (Figure 8C).

## 4. Discussion

The growth of *D. officinale* was not affected significantly by the salt stress for 14 days of treatment. In addition, the water content of leaves decreased but did not show significant difference with the control after two weeks’ exposure to salt stress. It might be due to the slow growth and salt tolerance of this species. In this study, we performed a transcriptomic and metabolomic analysis of *D. officinale* exposed to high salt concentration and investigated to molecular mechanisms of *D. officinale* leaves in response to salt stress.

### 4.1. The Molecular Mechanisms of D. officinale Leaves in Response to Salt Stress

Sugars (such as sucrose, glucose, trehalose, and fructose) with osmotic protection, carbon storage, and ROS scavenging abilities accumulate under salt stress and are related to salt tolerance [26,27]. As a molecular stabilizer, trehalose may prevent water loss and biomolecules denaturing during dehydration [28]. The accumulation of trehalose plays a role in osmotic protection when plants are exposed to salinity stress [12]. T6P is produced by the binding of a glucose 6-phosphate and a UDP-glucose under the catalysis of trehalose phosphate synthase, and is cleaved into trehalose by trehalose phosphate phosphatase [29]. In this study, the content of T6P and trehalose increased under salt stress (Figure 5A and Table S5), suggesting that trehalose may be involved in ROS scavenging. As a ROS scavenger, raffinose and galactinol may mitigate oxidative damage under abiotic stress conditions. For instance, maize/corn (*Zea mays*) *RAFFINOSE SYNTHASE* (*ZmRAFS*) enhances plant drought tolerance through either the synthesis of more raffinose or by generating more myo-inositol via hydrolysis of galactinol [30]. Raffinose and myo-inositol accumulated significantly under salt stress (Figure 5A), suggesting that they may enhance the tolerance of *D. officinale* seedlings to salt stress. AsA, as a strong and active antioxidant, could help plants scavenge ROS to combat environmental stress [31]. Therefore, AsA enhanced the tolerance of *D. officinale* seedlings to salt stress by scavenging ROS. The biosynthesis of AsA in plants is mainly accomplished through the d-mannose/l-galactose pathway. The key enzymes involved in this pathway include GMP, GME, GGP, l-galactose-1-phosphate phosphatase (GPP), l-galactose dehydrogenase (GDH), and GLDH [32]. The GMP from *D. officinale* is involved in the biosynthesis of mannan. The content of mannose in the *35S:DoGMP* transgenic *A. thaliana* plants is higher than that in the wild type, thereby enhancing the tolerance of *A. thaliana* plants to salt stress [33]. The relative expression level of some *CELLULOSE SYNTHASELIKE A* (*CSLA*) genes which are involved in the synthesis of mannan in *D. officinale* is upregulated (Figure 6B), suggesting that the content of mannan in the leaves of *D. officinale* may increase under salt stress. The accumulation of AsA in High-Pigment-1 tomato is positively correlated with the expression level of *GMP* and *GME*, but negatively correlated with the expression level of *GLDH* [31]. In this study, genes such as *GMP*, *GME*, *GLDH*, and *CSLA* were likely to be transcriptionally regulated, modifying the final concentration of AsA in *D. officinale* leaves under salt stress, so that the AsA content did not increase significantly under salt stress.

The overproduction of ROS under salt stress triggers lipid peroxidation, harms photosynthetic pigments, and inhibits the photosynthetic rate. The AsA-GSH cycle can alleviate oxidative damage by scavenging excess ROS under abiotic stress [34]. The key enzymes involved in the AsA-GSH cycle in plants include ascorbate peroxidase (APX), glutathione reductase (GR), glutathione *S*-transferase (GST), and glutathione peroxidase (GPX). Overexpression of *A. thaliana* APX in tobacco (*Nicotiana tabacum*) chloroplasts reduces the toxicity of H_2_O_2_, thus enhancing tolerance to salt stress [35]. Tobacco plants overexpressing soybean (*Glycine soja*) *GST* show a higher tolerance at the seedling stage than wild-type plants to salt [36]. Overexpression of *Triticum aestivum GPX* confers strong tolerance to salt in *A. thaliana* [37]. The expression of the gene encoding *Oryza sativa* glutathione reductase 3 (OsGR_3_) is induced by salt stress [38]. Moreover, the *OsGR_3_* knockout mutant (*gr_3_*) is sensitive to salt stress, which indicates that GR_3_ is crucial for rice salt tolerance [39]. In our study, the expression levels of these genes were significantly upregulated under salt stress (Figure 7), indicating that they participated in the response of *D. officinale* to salt stress, thereby alleviating the inhibitory effect of stress on the growth of *D. officinale* plants to a certain extent.

### 4.2. Salt Stress Induces the Accumulation of Bioactive Compounds in D. officinale Leaves

Jasmonates (JAs), such as methyl jasmonate (MeJA), are described as small signaling molecules in plants, and are commonly used to induce gene expression involved in plant stress responses and enhance production of secondary metabolites [40]. Some studies have used MeJA to promote the accumulation of bioactive compounds (polysaccharides, alkaloids, and flavonoids) in protocorm-like bodies of *D. officinale* [41,42]. JA accumulates within less than 120 s in wounded *Arabidopsis* leaves [43]. After 2 h of salt-stress treatment, the JA content in *D. officinale* leaves also increased significantly (Figure 8A). When plants are exposed to environmental stress, they generate JAZ-Ile at concentrations as low as 50 nM, which significantly promotes the interaction between COI1 and JAZ proteins [44]. Afterwards, JAZs subject to degradation by the 26S proteasome, and the released transcription factors (TFs) activate the expression of JA-responsive genes [45]. Flavonoids are valuable natural products derived from the phenylpropanoid pathway [46]. The MBW complex formed by the interaction R2R3-MYB, basic helix-loop-helix (bHLH) and WD40 proteins usually regulates gene expression involved in the flavonoid biosynthesis pathway [47,48]. In our results, the contents of Ile and JA increased significantly under salt stress (Figure 5A and Figure 8A), and the expression level of *DobHLH* (*gene9655*) (a homolog of *AtMYC4*) and *DoMYB* (*gene6015*) (a homolog of *AtMYBTT2*) were downregulated and upregulated, respectively, under salt stress. Furthermore, salt stress induced the accumulation of flavonoids such as rutin in *D. officinale* leaves (Figure 5B). Hence, we hypothesized that the interaction between JAZ and DobHLH (gene9655) would inhibit JA response under normal circumstances. In contrast, salt stress could trigger JA biosynthesis, and synthetic JA-Ile would induce the interaction of COI1 with JAZ proteins, leading to the degradation of JAZ proteins via the 26S proteasome, and DoMYB (gene6015) was released to activate the expression of genes involved in flavonoid biosynthesis (Figure S7). Therefore, cultivating *D. officinale* plants in saline-alkali soil may be beneficial to increase the content of secondary metabolites (such as flavonoids) in leaves.

## 5. Conclusions

We carried out transcriptome and metabolite profiling to illuminate the response mechanisms of *D. officinale* leaves under salt stress. The DEGs related to the biosynthesis pathways of JA and flavonoids in *D. officinale* leaves have been identified. The results demonstrated that salt stress could trigger JA biosynthesis, and JA, which as a signal molecule, further promotes flavonoid biosynthesis. Therefore, for *D. officinale* plants, the application of exogenous MeJA may increase the content of secondary metabolites such as flavonoids in leaves. In addition, salt stress also increased the contents of other compounds, such as sugars and alkaloids, in *D. officinale* leaves. The accumulation of these compounds, which have strong health benefits, may play an imperative role in how *D. officinale* plants adapt to salt stress. These results could help to illustrate physiological response and molecular mechanisms in an important orchid plant *D*. *officinale* under salinity stress and help to understand the relationship between quality and yield.

## Data Availability

National Centre for Biotechnology Information, https://www.ncbi.nlm.nih.gov/bioproject/?term=PRJNA715099, accessed on 1 March 2021.

## Supplementary Materials

The following are available online at https://www.mdpi.com/article/10.3390/biom11050736/s1, Figure S1: Number of differentially expressed transcription factors (TFs) between salt stress and control treatments. (**A**) Number of both up- and downregulated TFs. (**B**) Number of upregulated TFs. (**C**) Number of downregulated TFs, Figure S2: The expression profile of bHLH transcription factor family genes and PPI networks. (**A**) Heat map analysis of gene expressions under salt stress. (**B**) The predicted PPI of DEGs obtained from the protein–protein interaction in the STRING database. (**C**) PPI networks of *A. thaliana* genes homologous to *DobHLH* (*gene9655*), Figure S3: The expression profile of TIFY transcription factor family genes at different time points under salt stress, Figure S4: The expression profile of MYB transcription factor family genes and PPI networks. (**A**) Heat map analysis of gene expressions under salt stress. (**B**) The predicted PPI of DEGs obtained from the protein–protein interaction in the STRING database. (**C**) PPI networks of *A. thaliana* genes homologous to *DoMYB* (*gene6015*), Figure S5: Neighbor-joining phylogenetic tree showing the relatedness of the amino acid sequences of the *D. officinale* MYB-related protein (accession number: XP 020672817.1) and 122 MYB-related proteins from *A. thaliana*. The MYB-related protein from *D. officinale* is indicated with a red triangle. ‘At’ is an abbreviation for *A. thaliana*. The neighbor-joining phylogenetic tree was constructed using the MEGA 7 program with a bootstrap analysis of 1000 replicates. The accession numbers and amino acid sequences of the MYB-related protein from *D. officinale* and 122 MYB-related proteins from *A. thaliana* are shown in Table S6, Figure S6: PCA score plots (**A**), types (**B**), and KEGG pathway annotations of metabolites in leaves (**C**). ‘Total’ refers to the total types of identified metabolites. ‘Up’ and ‘Down’ refer the types of metabolites whose content is significantly increased and decreased, respectively, Figure S7: Putative simplified model of JA signaling in the biosynthesis of flavonoids. (**A**) JAZ and DobHLH (gene9655) interact to inhibit the response of JA under normal conditions. (**B**) Salt stress could trigger JA-Ile biosynthesis. Perception of JA-Ile by COI1 leads to proteasomal degradation of the repressor JAZ and, simultaneously, transcription factor DoMYB (gene6015) is released to activate the expression of downstream genes promoting flavonoid biosynthesis, Table S1: Primer sequences used in qRT-PCR, Table S2: Number and quality of RNA-Seq reads produced in each sample, Table S3: The result of DobHLH (gene9655) sequence being blasted to the Plant Transcription Factor Database, Table S4: The result of DoMYB (gene6015) sequence being blasted to the Plant Transcription Factor Database, Table S5: Major metabolites detected by using MRM (Multiple Reaction Monitoring) based on novogene in-house database. FC represents ratio of level in treatment to control. *p* values were calculated by univariate analysis (*t*-test), Table S6: The accession numbers and amino acid sequences of MYB-related protein from *D. officinale* and 122 MYB-related proteins from *A. thaliana*.
