# Supplementary material for: Transcriptome and Metabolome Reveal Salt-Stress Responses of Leaf Tissues from Dendrobium officinale
Source: Biomolecules. 2021 May 15;11(5):736. doi: 10.3390/biom11050736 (PMC8156352; doi:10.3390/biom11050736)
Supplement: Supplementary file 1 [file biomolecules-11-00736-s001.zip › Table S1.pdf]

**Table S1** Primer sequences used in qRT-PCR.

| Primer name | Sequence (5′ to 3′) |
|---|---|
| Actin-F | TCCCAAGGCAAACAGAGAAA |
| Actin-R | GGCCACTAGCATATAGGGAAAG |
| DoANS-F | CTCCTTACTCTCTCTCGGTCTC |
| DoANS-R | CAGCGTGGGTAGTAGTTGATTT |
| DoAOC-F | CCTAAGCCAGAAGGAAGTCAAT |
| DoAOC-R | CTGTTATTCCGAGCCTCTTCTC |
| DoAOS-F | GTTCCGAAGCTCGTTGAAGA |
| DoAOS-R | TCCAGCCGTGTTGAAGTAATC |
| DoCHI-F | CGTCTACACCGAAGAAGAAGAAG |
| DoCHI-R | GGAATGAGAGAAGACGATGGAAG |
| DoF3′5′H-F | GGGCTTCATCTCTGACCTATTC |
| DoF3′5′H-R | GTCGGAGGATTGATGGGTTT |
| DoLOX2S-F | AGATTCCGGAGATGATGGAAAG |
| DoLOX2S-R | CAGCTGGATACTGAGAGGATTG |
| DoMFP-F | AAGTTGGAGGATGCGTATGG |
| DoMFP-R | GCTTGCGAGAGAGATGAATTTG |
| DoOPR-F | TCCTCTCACATCGGGTAGTT |
| DoOPR-R | GTCAGTGGCTCTTTGGGTATAG |
