# Supplementary material for: Transcriptome and Metabolome Reveal Salt-Stress Responses of Leaf Tissues from Dendrobium officinale
Source: Biomolecules. 2021 May 15;11(5):736. doi: 10.3390/biom11050736 (PMC8156352; doi:10.3390/biom11050736)
Supplement: Supplementary file 1 [file biomolecules-11-00736-s001.zip › Table S2.pdf]

Table S2 Number and quality of RNA-seq reads produced in each sample. Sample 1, Sample 2, and Sample 3 indicate three biological replicates.

Sample	Total Reads (M)	Total Clean Reads (M)	Total Clean Bases (Gb)	GC Content (%)	Clean Reads≥ Q30 (%)	Mapped Reads (%)
Sample_1_0 h	58.80	29.40	8.78	48.78	93.61	91.27
Sample_2_0 h	41.21	20.61	6.17	48.47	93.59	91.17
Sample_3_0 h	46.88	23.44	7.00	48.40	93.69	91.29
Sample_1_4 h	63.03	31.51	9.40	48.47	93.50	91.08
Sample_2_4 h	42.45	21.23	6.33	48.45	93.51	91.04
Sample_3_4 h	53.02	26.51	7.91	48.34	93.85	91.08
Sample_1_12 h	52.98	26.49	7.90	47.84	93.27	91.07
Sample_2_12 h	54.71	27.35	8.15	47.67	93.75	91.52
Sample_3_12 h	41.07	20.54	6.14	47.50	93.80	91.36
