# Supplementary material for: Transcriptome and Metabolome Reveal Salt-Stress Responses of Leaf Tissues from Dendrobium officinale
Source: Biomolecules. 2021 May 15;11(5):736. doi: 10.3390/biom11050736 (PMC8156352; doi:10.3390/biom11050736)
Supplement: Supplementary file 1 [file biomolecules-11-00736-s001.zip › Table S3.pdf]

Table S3 The result of the protein (Gene9655) sequence being blasted to the Plant Transcription Factor Database.

Query: DoMYC2(XP_020675326.1)

Hit ID	Description	Score	E-Value
AT4G17880.1	Arabidopsis thaliana bHLH family protein	438	4e-49
AT1G32640.1	Arabidopsis thaliana bHLH family protein	436	1e-48
AT5G46760.1	Arabidopsis thaliana bHLH family protein	384	1e-41
AT4G16430.1	Arabidopsis thaliana bHLH family protein	344	1e-36
AT4G00870.1	Arabidopsis thaliana bHLH family protein	322	7e-34
AT5G46830.1	Arabidopsis thaliana NACL-inducible gene 1	318	7e-33
AT2G46510.1	Arabidopsis thaliana ABA-inducible BHLH-type transcription factor	283	4e-28
AT1G01260.1	Arabidopsis thaliana bHLH family protein	261	3e-25
AT1G01260.3	Arabidopsis thaliana bHLH family protein	261	3e-25
AT1G01260.2	Arabidopsis thaliana bHLH family protein	261	3e-25
AT4G09820.1	Arabidopsis thaliana bHLH family protein	198	2e-17
AT4G00480.2	Arabidopsis thaliana bHLH family protein	173	3e-14
AT4G00480.1	Arabidopsis thaliana bHLH family protein	172	3e-14
AT5G41315.1	Arabidopsis thaliana bHLH family protein	172	3e-14
AT1G63650.1	Arabidopsis thaliana bHLH family protein	172	4e-14
AT1G63650.2	Arabidopsis thaliana bHLH family protein	172	4e-14
AT1G63650.3	Arabidopsis thaliana bHLH family protein	172	4e-14
AT4G37850.1	Arabidopsis thaliana bHLH family protein	158	8e-13
