# Supplementary material for: Transcriptome and Metabolome Reveal Salt-Stress Responses of Leaf Tissues from Dendrobium officinale
Source: Biomolecules. 2021 May 15;11(5):736. doi: 10.3390/biom11050736 (PMC8156352; doi:10.3390/biom11050736)
Supplement: Supplementary file 1 [file biomolecules-11-00736-s001.zip › Table S4.pdf]

Table S4 The result of the protein (Gene6015) sequence being blasted to the Plant Transcription Factor Database.

Query: DoMYB114-XP_020672817.1

Hit ID	Description	Score	E-Value
AT5G35550.1	Arabidopsis thaliana MYB family protein	453	7e-57
AT4G38620.1	Arabidopsis thaliana myb domain protein 4	452	2e-56
AT1G22640.1	Arabidopsis thaliana myb domain protein 3	449	3e-56
AT2G16720.1	Arabidopsis thaliana myb domain protein 7	435	5e-54
AT4G34990.1	Arabidopsis thaliana myb domain protein 32	430	4e-53
AT5G14750.1	Arabidopsis thaliana myb domain protein 66	423	5e-53
AT4G09460.1	Arabidopsis thaliana myb domain protein 6	423	1e-52
AT3G13540.1	Arabidopsis thaliana myb domain protein 5	424	1e-52
AT5G60890.1	Arabidopsis thaliana myb domain protein 34	421	1e-51
AT5G40330.1	Arabidopsis thaliana myb domain protein 23	415	1e-51
AT1G35515.1	Arabidopsis thaliana high response to osmotic stress 10	406	3e-50
AT3G23250.1	Arabidopsis thaliana myb domain protein 15	409	5e-50
AT5G26660.1	Arabidopsis thaliana myb domain protein 86	414	6e-50
AT5G61420.2	Arabidopsis thaliana myb domain protein 28	415	6e-50
AT5G16770.2	Arabidopsis thaliana myb domain protein 9	412	9e-50
AT5G16770.1	Arabidopsis thaliana myb domain protein 9	412	9e-50
AT5G52600.1	Arabidopsis thaliana myb domain protein 82	400	1e-49
AT5G07690.1	Arabidopsis thaliana myb domain protein 29	411	1e-49
AT4G05100.1	Arabidopsis thaliana myb domain protein 74	409	2e-49
AT4G28110.1	Arabidopsis thaliana myb domain protein 41	404	3e-49
AT4G01680.3	Arabidopsis thaliana myb domain protein 55	401	3e-49
AT3G02940.1	Arabidopsis thaliana myb domain protein 107	405	7e-49
