# Supplementary figures and images for: Transcriptome and Metabolome Reveal Salt-Stress Responses of Leaf Tissues from Dendrobium officinale
Source: Biomolecules. 2021 May 15;11(5):736. doi: 10.3390/biom11050736 (PMC8156352; doi:10.3390/biom11050736)

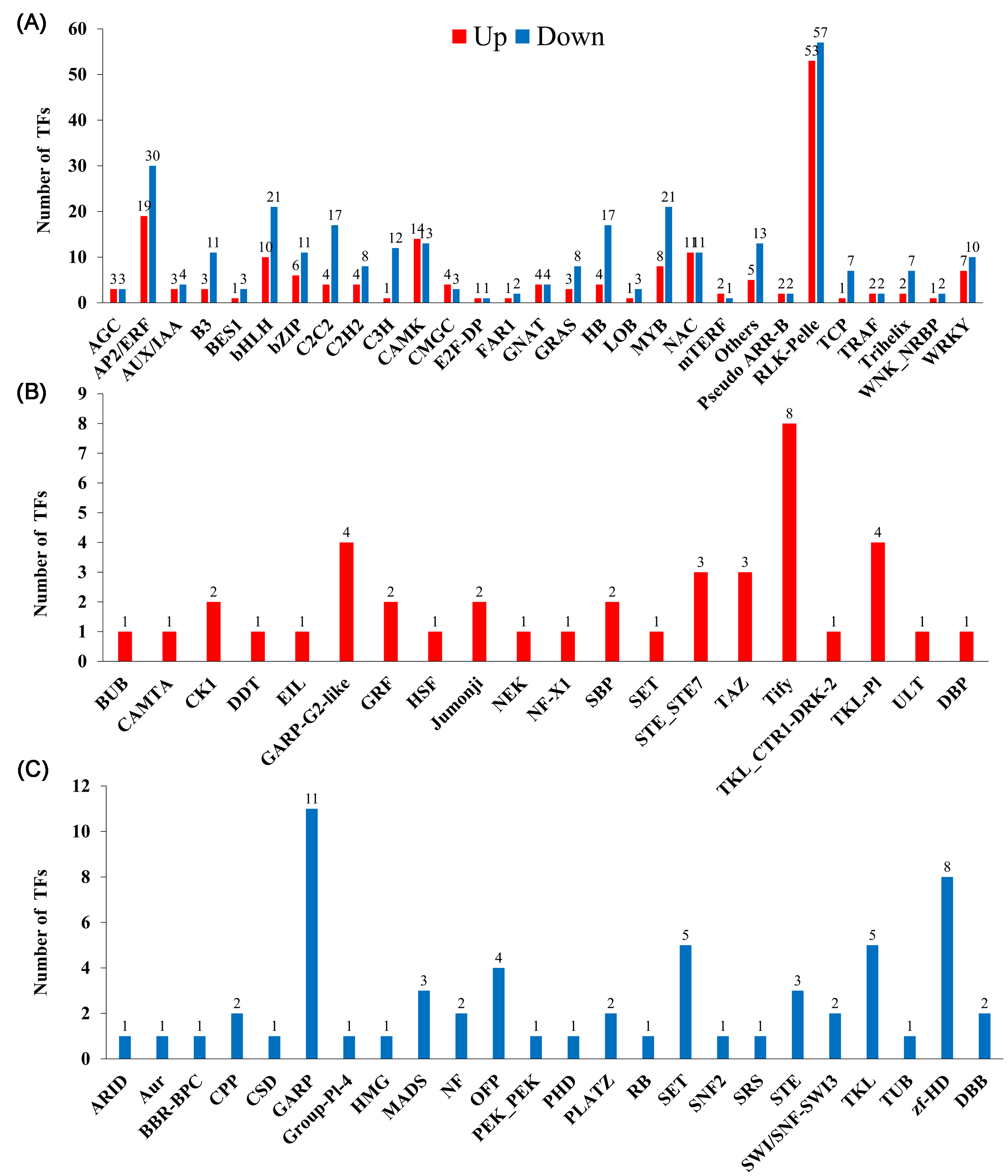

Supplement: Supplementary file 1 [file biomolecules-11-00736-s001.zip › Figure S1.tif]

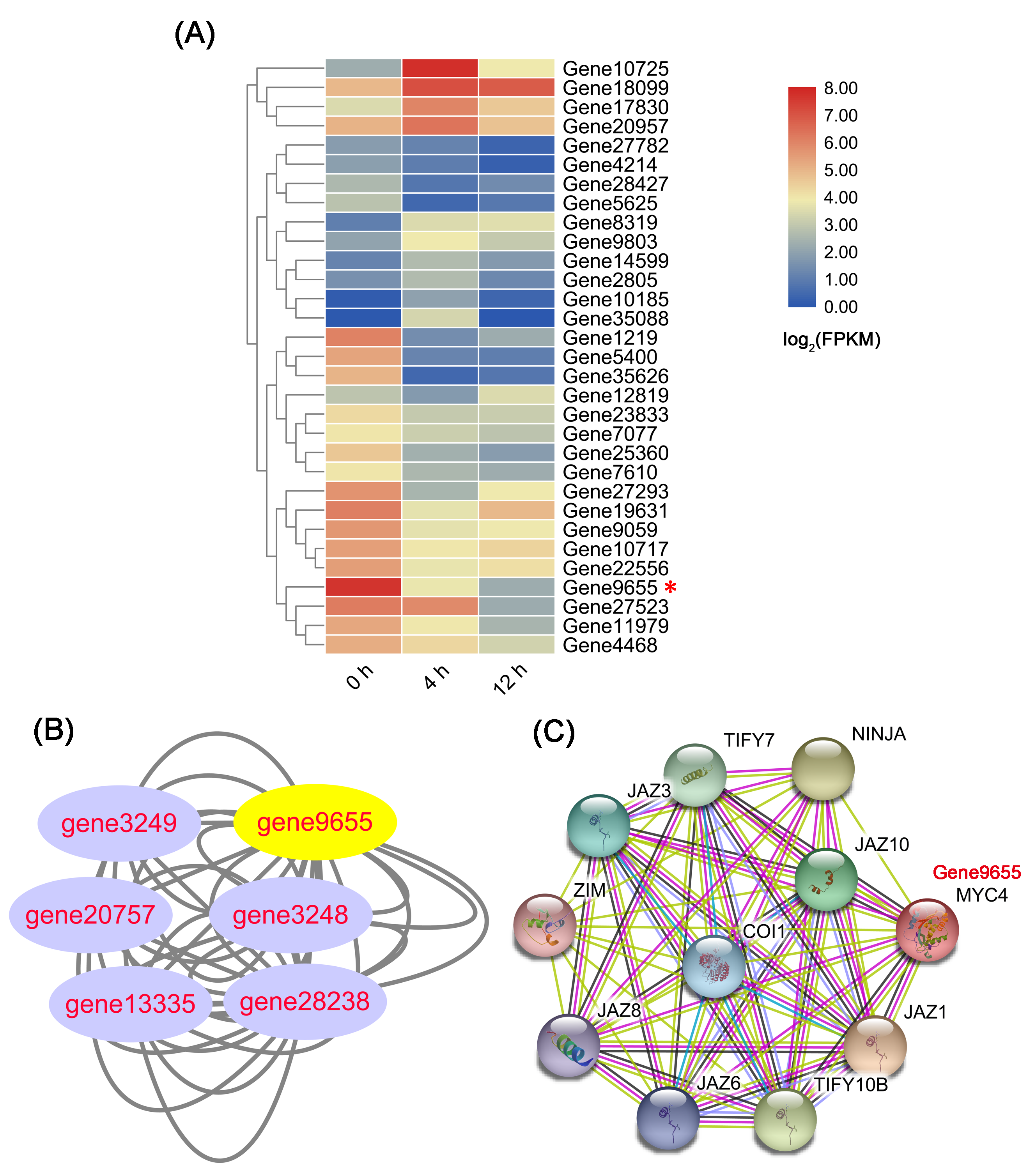

Supplement: Supplementary file 1 [file biomolecules-11-00736-s001.zip › Figure S2.tif]

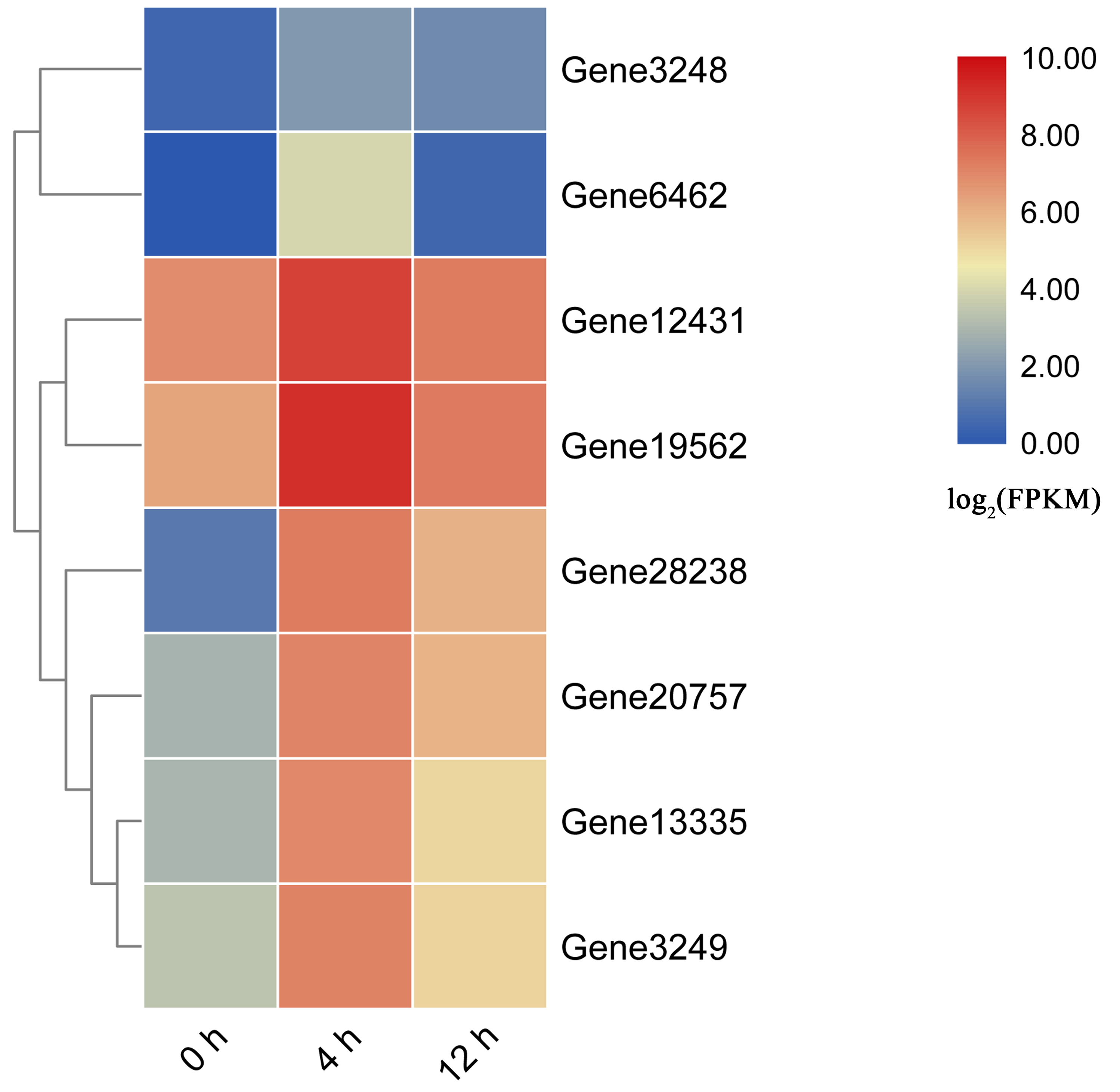

Supplement: Supplementary file 1 [file biomolecules-11-00736-s001.zip › Figure S3.tif]

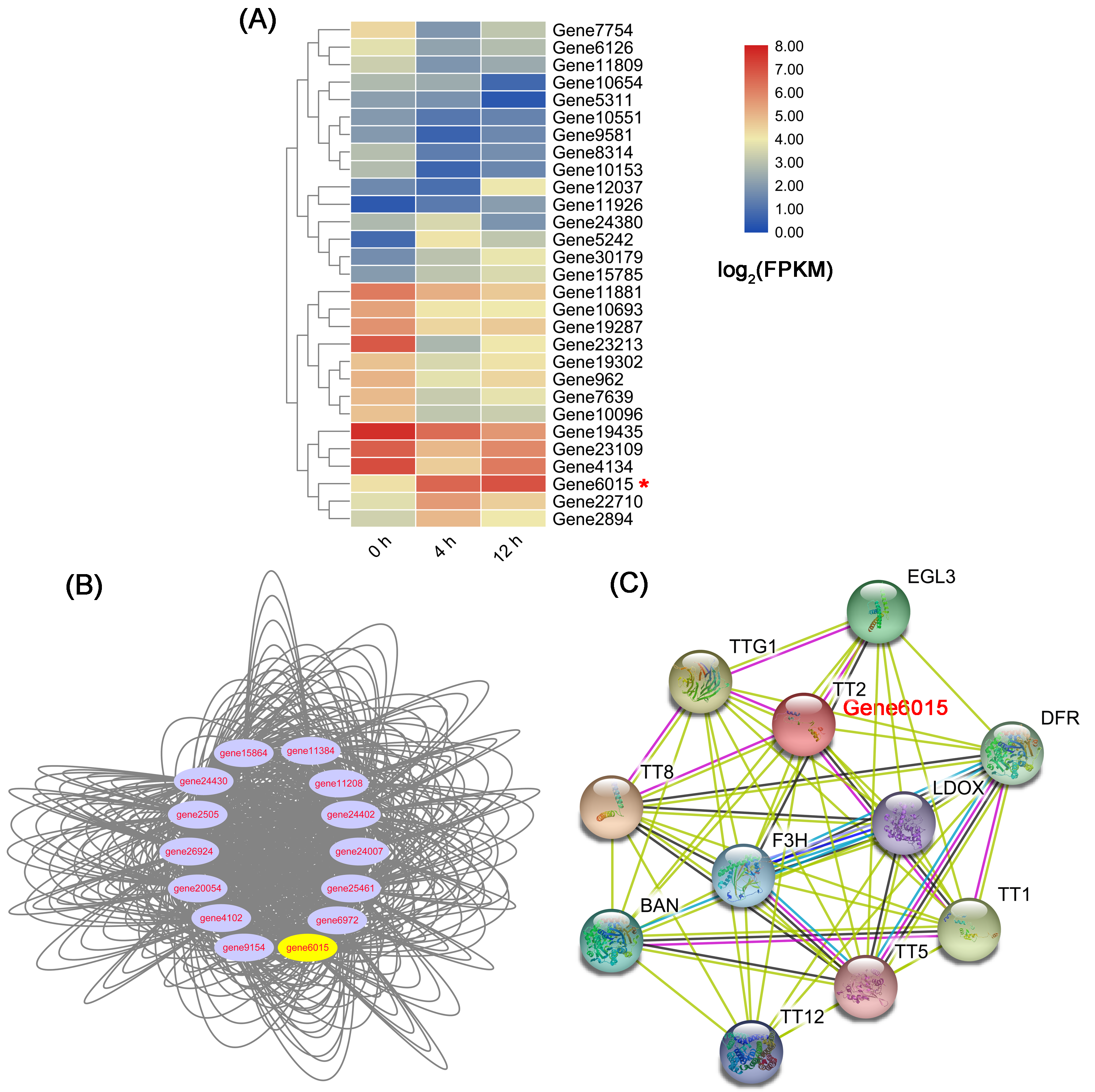

Supplement: Supplementary file 1 [file biomolecules-11-00736-s001.zip › Figure S4.tif]

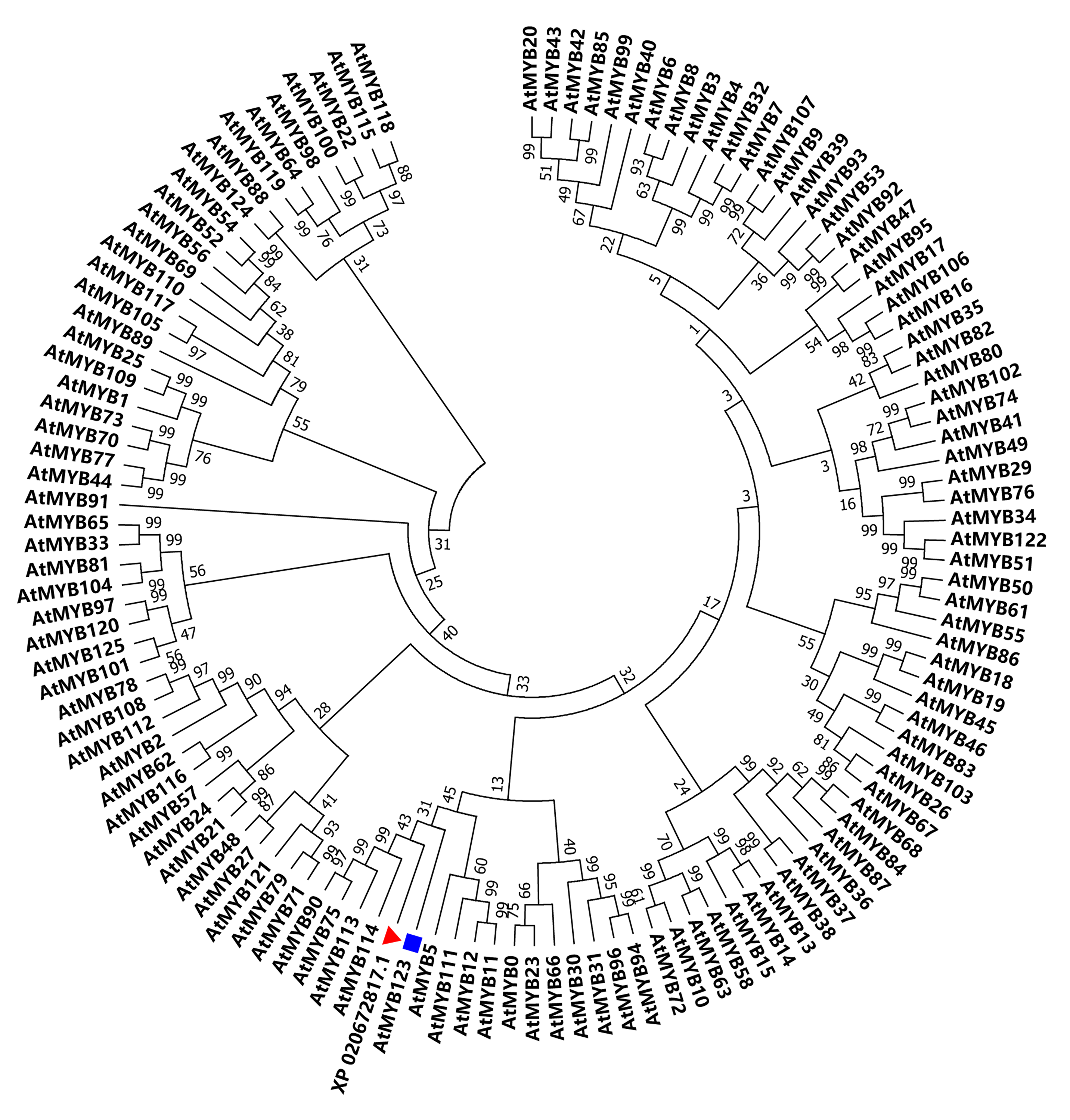

Supplement: Supplementary file 1 [file biomolecules-11-00736-s001.zip › Figure S5.tif]

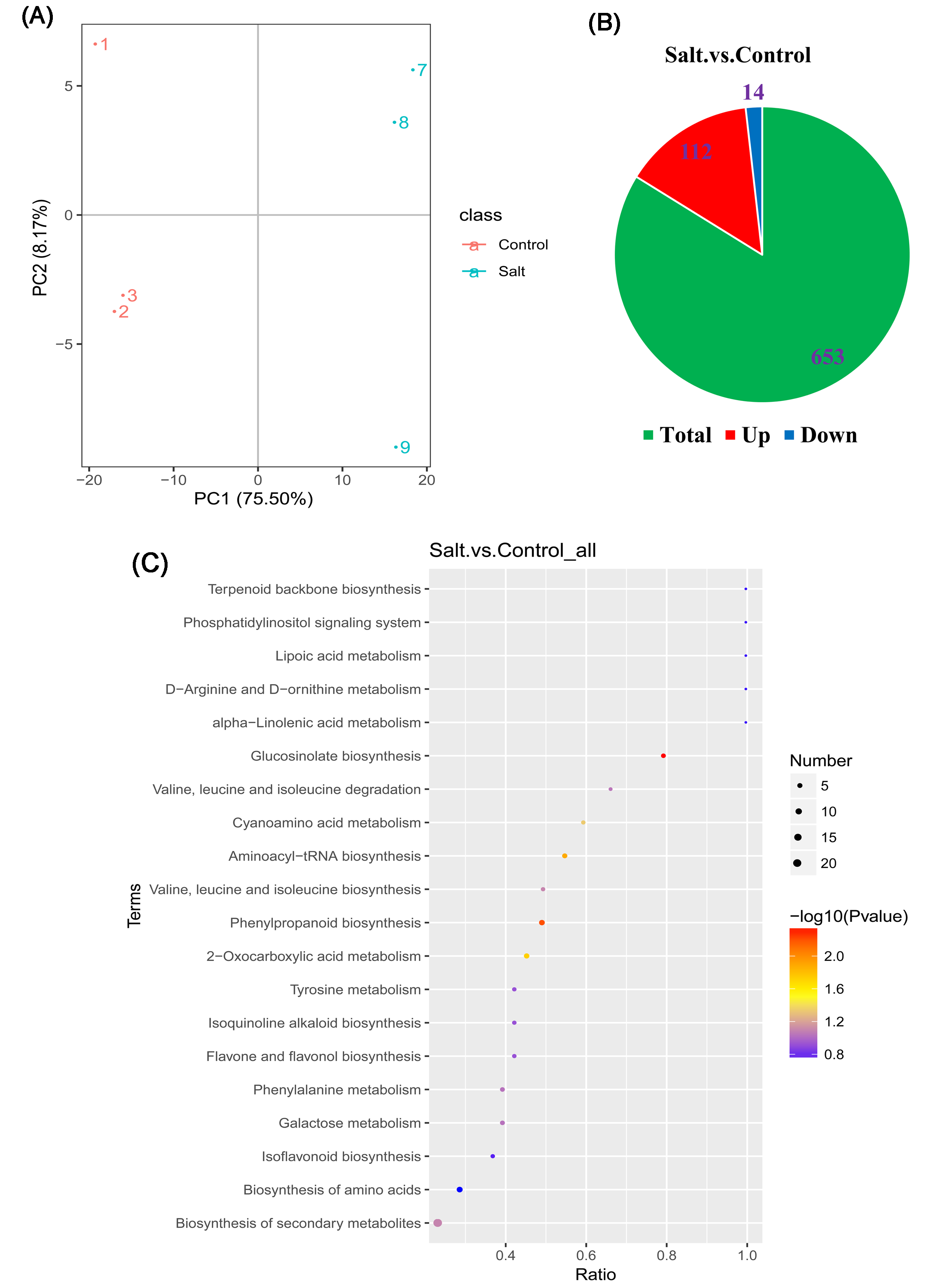

Supplement: Supplementary file 1 [file biomolecules-11-00736-s001.zip › Figure S6.tif]

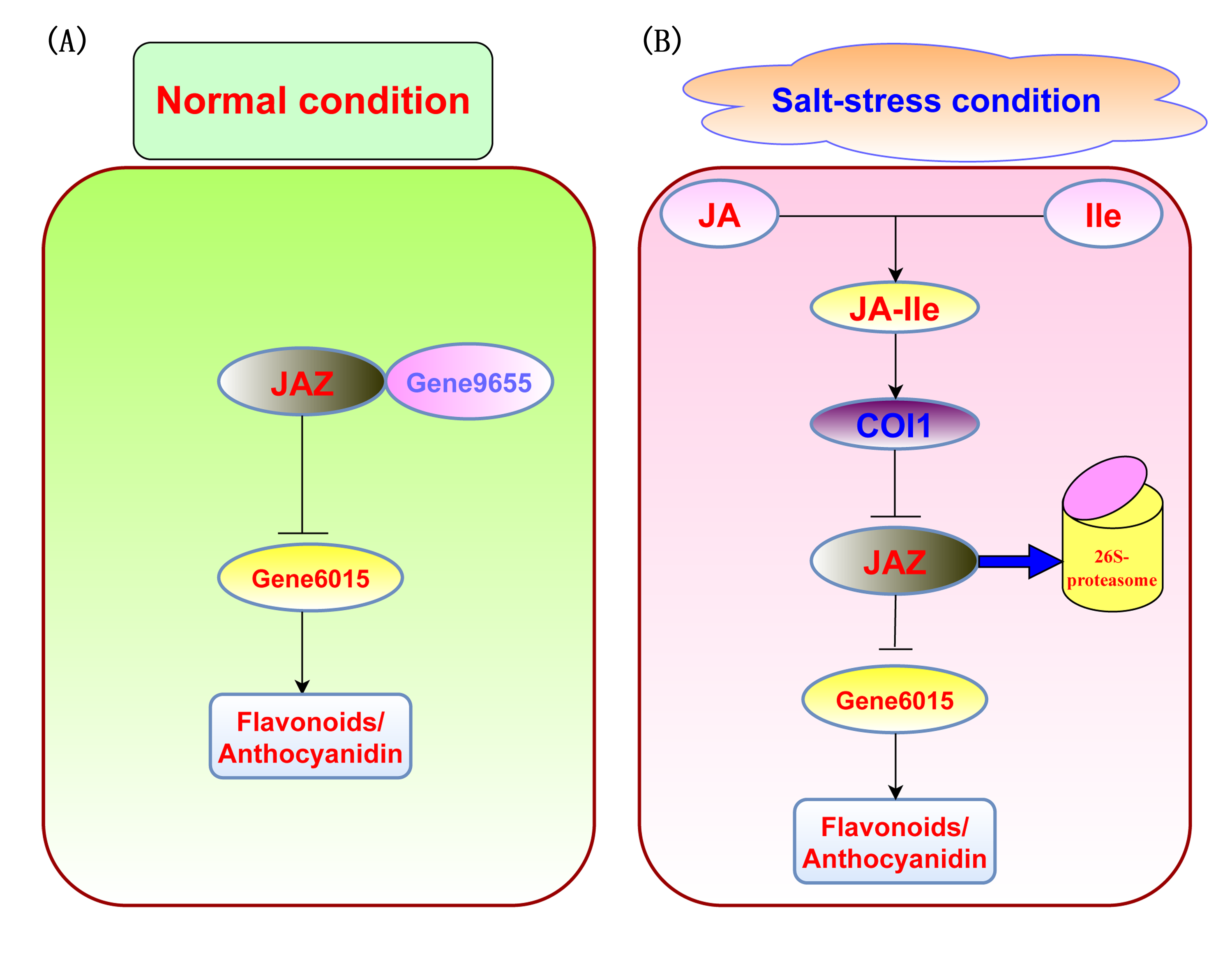

Supplement: Supplementary file 1 [file biomolecules-11-00736-s001.zip › Figure S7.tif]
